# How did the COVID-19 pandemic affect antibiotic consumption within humanitarian emergencies? Results from five humanitarian contexts

**DOI:** 10.1016/j.infpip.2024.100385

**Published:** 2024-07-18

**Authors:** Tuba Yavuz, Kate Clezy, Kristina Skender, Jacob Goldberg, Frédérique Vallières

**Affiliations:** a Trinity Centre for Global Health, Trinity College Dublin, Dublin, Ireland; b Operational Centre Amsterdam (OCA), Médecins Sans Frontières, Amsterdam, the Netherlands; c School of Psychology, Trinity College Dublin, Dublin, Ireland

**Keywords:** Antibiotic consumption, Time-series analysis, Humanitarian settings, COVID-19

## Abstract

**Introduction:**

Both high- and low-income countries reported increased antibiotic consumption among COVID-19 patients during the first months of the pandemic. To date, however, no studies have examined changes in antibiotic consumption during the COVID-19 pandemic within humanitarian emergency contexts.

**Method:**

Data was collected by Médecins Sans Frontières (MSF) for the years 2018–2021 across the following humanitarian settings: Afghanistan (Lashkar Gah), Bangladesh (Kutupalong), the Democratic Republic of Congo (Mweso and Baraka), and South Sudan (Bentiu). Inpatient and outpatient antibiotic consumption was calculated as Daily Defined Dose (DDD) per 1000 inhabitants per day, as per the World Health Organisation's (WHO) Collaborating Centre for Drug Statistics Methodology. Interrupted time series (ITS) analysis, using an autoregressive integrated moving average (ARIMA) model was used to analyse retrospective monthly antibiotic consumption. The impact of COVID-19 pandemic was evaluated as total antibiotic consumption and according to WHO Access, Watch, Reserve (AWaRe) group classifications within each humanitarian setting.

**Results:**

The COVID-19 pandemic had no statistically significant impact on total antibiotic consumption in South Sudan (Bentiu) and Bangladesh (Kutupalong). Similarly, the pandemic had no impact on total antibiotic consumption in DR Congo (Baraka), despite an initial 0.27% (estimate=.274, p-value=0.006) increase in March 2020 driven by Access group antibiotics. Meanwhile, total antibiotic consumption in DR Congo (Mweso) and Afghanistan (Lashkar Gah) declined by 0.74% (estimate = −.744, p = 0.003) and 0.26% (estimate = −.26, p < 0.001), respectively with the COVID-19 pandemic.

**Conclusion:**

Further studies are required to investigate what may have contributed to these results.

## Introduction

Antimicrobial resistance (AMR) represents one of the most urgent threats to global health security [1,2], undermining our ability to fight several multidrug-resistant infections [3]. A recent comprehensive systematic review assessing the global burden of antimicrobial resistance, including estimates from 204 countries and territories, found that the deaths associated with bacterial AMR in 2019 alone totalled 4.95 million, of which 1.27 million deaths were directly caused by AMR [4]. Left unaddressed, annual deaths due to AMR are expected to rise to 10 million deaths by 2050 [[4], [5], [6], [7]], at a cost of 100 trillion USD [7].

In low- and middle-income countries (LMICs), where the majority of humanitarian events occur [8,9], the burden of AMR is exacerbated by multiple factors. These include high rates of infectious diseases; poor water, sanitation, and hygiene practices (WASH); low vaccination coverage [3,10]; restricted or lack of access to essential and new line of antimicrobials [11,12]; and the overuse or misuse of antimicrobials due to inadequate antimicrobial stewardship (AMS) [10,13]. In addition, loosely structured regulations that ease the access to antimicrobials, and challenges around antibiotic seeking and consumption behaviours (i.e., self-medication, patient adherence to the treatment, influences on prescribing patterns including promotional activities of pharmaceutical companies, and patient pressure on doctors to prescribe antibiotics) are other factors that increase the burden of AMR [3,14,15]. Moreover, the emergence and spread of AMR are compounded by humanitarian crisis given the additional strain humanitarian events place on what are often weakened healthcare systems, operating with scarce human, material and economic resources [16,17]. Also, the humanitarian events might lead to disruption in vaccination programmes [18].

AMS is a set of actions and guidance/guidelines that promote appropriate use of antimicrobials. Specifically, AMS comprises a set of actions that aim to measure and improve the use of antimicrobials by guiding the selection of the optimal antimicrobial drug, dose, duration of treatment, and route of administration [19,20], and at the same time to ensure access to effective medicines for the populations in need [10]. AMS is an integral part of the response to AMR [4,21], however, the literature on AMS interventions in LMICs is relatively limited, and several challenges including, scarce resources in trained staff, diagnostic, medicines and inadequate policies/programmes for implementation of AMS have been reported for these countries [10,13]. Médecins Sans Frontières (MSF) has commenced antibiotic stewardship programmes in humanitarian settings as an attempt to address AMR. They were first implemented within a surgical hospital in Amman, Jordan which found them to be acceptable and effective [22,23]. MSF also trains its medical staff about antibiotic prescription approaches and attitudes in the context of antibiotic stewardship [24].

Moreover, surveillance of antimicrobial resistance and consumption is important to identify the scope of problem, establish interventions that target irrational/inappropriate use of antimicrobials, and decrease pathogen resistance selection pressure [25]. To this end, a number of systems and policies have been put in place to survey and monitor AMR across settings (e.g., the European Antimicrobial Surveillance System Network (EARS-Net) [3], the Global Action Plan to tackle AMR (GAP-AMR), and the Global Antimicrobial Resistance and Use Surveillance System (GLASS)) [26]. Developed by the World Health Organisation (WHO), the Access, Watch, and Reserve (AWaRe) system was established to promote rational use of antibiotics based on their resistance potential and preference for use [27].

The overuse of antibiotics during the COVID-19 pandemic, particularly in the first wave or first months [28], threatened to undermine on-going efforts to address AMR. Early concerns about bacterial co-infections and secondary bacterial infections led to an increase in antibiotic prescription during the COVID-19 pandemic, particularly in the first wave, when the knowledge and experience of treating the disease was still in its preliminary phase [29,30]. Increases in antibiotic use within acute care settings were observed in several countries including Spain [31,32], Italy [33], Ireland [34], and in the Netherlands [35]. Increases in community antibiotic consumption were also reported in India [36]. On the other hand, Israel [37] and New Zealand [38] reported a decrease in antibiotic consumption during COVID-19 pandemic. More recently, Khouja's study [39] reported on the antibiotic consumption patterns of 66 countries during the COVID-19 pandemic and found that despite an initial increase in antibiotic consumption, most countries showed an eventual decrease from April to August 2020.

Comparatively, however, little is known about how antibiotic consumption fared within humanitarian contexts during the COVID-19 pandemic [17], which, in many contexts, caused AMR surveillance to pause [16,17,40]. Indeed, and to the best of our knowledge, no studies to date have investigated changes in antibiotic consumption during the COVID-19 pandemic within humanitarian emergencies. The purpose of this study was therefore to understand whether, and if so how, the COVID-19 pandemic affected antibiotic consumption within a wide geographical range of humanitarian contexts.

## Methods

### Research settings

The research settings in this study were the secondary healthcare hospitals supported by MSF in Afghanistan (Lashkar Gah), Bangladesh (Kutupalong), DR Congo (Mweso and Baraka), and South Sudan (Bentiu) humanitarian emergencies. These are the only large secondary healthcare hospitals within the geographical areas of the humanitarian settings. MSF provides medical and humanitarian aid across five regions in Afghanistan including a secondary healthcare hospital in Lashkar Gah that serves a catchment area of close to two million people across all medical departments. The hospital receives a considerably high number of patients particularly in malnutrition, infectious diseases, maternal health and surgery [41,42].

MSF's Kutupalong hospital in Bangladesh's Cox's Bazar district serves approximately eighteen thousand Rohingya refugees and also the local community [43]. The hospital treats a wide range of diseases including respiratory infections, diarrhoea which are commonly linked to poor WASH practices, and also infectious diseases remain a concern among the settlements of the refugees [44].

Furthermore, MSF works on large-scale projects in DR Congo to respond to the immense needs of the population. MSF-supported Baraka hospital in South Kivu provided secondary healthcare to a catchment area of over one million people in Fizi territory [45] that was mainly affected from malaria, diarrhoea, and respiratory tract infections. However, MSF stopped supporting Baraka hospital in 2021 due to security reasons [46]. On the other hand, MSF-supported Mweso hospital in North Kivu province provides secondary healthcare, and serves a catchment area of almost half million people [47]. The hospital provides treatment for malaria, HIV, tuberculosis, malnutrition, respiratory infections and diarrhoeal diseases [48].

**Figure 1:**
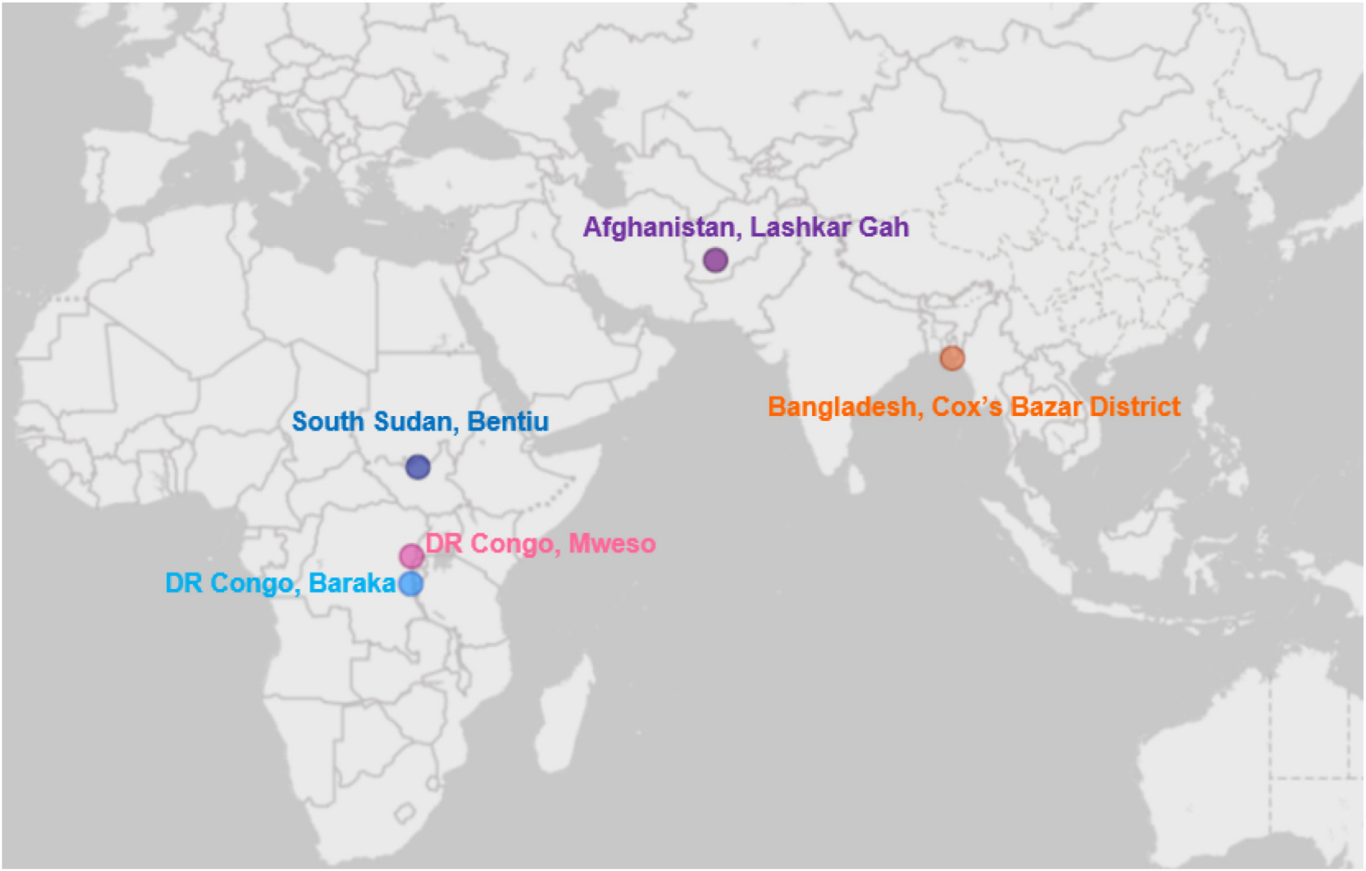
Geographic locations of research settings.

Considering the last research setting, MSF's hospital in Bentiu, South Sudan serves a catchment area of more than 100,000 people [49]. The hospital provides specialist healthcare, surgery and emergency services, and also treatment of infectious diseases [50]. Geographic locations of MSF-supported hospitals are shown in Figure 1.

### Data collection

Data on antibiotic consumption of inpatient and outpatient adults were collected via MSF developed tools for collection of health information and consumption of medical items; District Health Information System Version 2.0 (DHIS-2) and Consumption Tool (CT), respectively across the five humanitarian settings: Afghanistan (Lashkar Gah), Bangladesh (Kutupalong), DR Congo (Mweso and Baraka), and South Sudan (Bentiu), for pre-pandemic (2018, 2019, and January and February of 2020) and pandemic (March 2020 onwards and 2021) periods. MSF shared irrevocably anonymised collected data with the researcher in the Trinity Centre for Global Health between 25^th^ and 29^th^ of July 2022.

### Data analysis

Antibiotic consumption included both inpatient and outpatient consumption data, reported as a monthly total, and WHO AWaRe antibiotic group for each humanitarian setting, for the years 2018–2021. Daily Defined Dose (DDD) using the Anatomical Therapeutic Chemical Classification System (ATC/DDD) developed by the WHO Collaborating Centre for Drug Statistics Methodology was used to measure antibiotic consumption across all settings. Data were analysed using interrupted time series (ITS), and more specifically using an ARIMA model. Given the presence of seasonality and autocorrelation, an ARIMA model was estimated using maximum likelihood. Specifically, ARIMA forecasts antibiotic consumption in the absence of the pandemic (i.e., the “counterfactual”) and then determines how the observed diverges from this forecast. March 2020 was selected as the point of interruption, corresponding to the month that the WHO officially declared COVID-19 a pandemic [51]. A “pulse” intervention in the first month (March 2020) of the pandemic was fitted. In addition, the impact of COVID-19 pandemic in the first months (April–August 2020) was evaluated by a “ramp” function. Finally, the overall impact of COVID-19 pandemic interruption was examined by using a “step” function. Recommended steps by Schaffer's study [52] were then followed to identify the best ARIMA model for the analysis. IBM SPSS Statistics (Version 27) was used to conduct all the analyses.

### Steps to identify parameters for ARIMA model

First, all the data was plotted to understand emerging patterns as ARIMA modelling in time series requires stationary series, or observations. No log-transformation was applied to the data. Next, and where an observed linear trend and seasonality were observed, and in order to induce stationarity, a first order differencing (*d*=1) was applied. Seasonal differencing (D=1) was only applied, where visually deemed necessary. The autocorrelation function (ACF)/partial autocorrelation function (PACF) was then used to examine the correlation between each observation and previous values at maximum 24 lags. Finally, Dixon's [53] ARIMA model resources were used and statistical parameters of the models (i.e., low normalized BIC value and non-significant Ljung-Box p-value) were checked to compare the selected AR/MA orders [52,53]. In addition, simple models were preferred to avoid over parameterisation [53]. The SPSS “Expert Modeler” function was only used to compare possible fitting ARIMA models.

### Ethical considerations

Ethical approval for this study was obtained from the Health Policy & Management/Centre for Global Health Research Ethics Committee, Trinity College Dublin and MSF Research Review Committee. A data sharing agreement was put in place between the Trinity Centre for Global Health and MSF.

## Results

Total antibiotic consumption as DDD per 1000 inhabitants per day, for the five settings are presented in Table I. DR Congo (Baraka) had the lowest and Bangladesh (Kutupalong) had the highest total DDD per 1000 inhabitants per day during the study period.

**Table I:** Total antibiotic consumption (DDD per 1000 inhabitants per day) across five humanitarian settings between 2018-2021

Year	Afghanistan (Lashkar Gah)	Bangladesh (Kutupalong)	DR Congo (Baraka)^a^	DR Congo (Mweso)	South Sudan (Bentiu)
2018	6,83	382,63	9,30	26,58	58,49
2019	9,02	312,19	6,96	29,38	38,80
2020	7,79	341,96	6,55	23,25	53,49
2021	9,46	403,20	1,34	30,80	57,85
Mean	8,28	360,00	6,04	27,50	52,16

a The setting had only antibiotic consumption data until April 2021.

**Figure 2:**
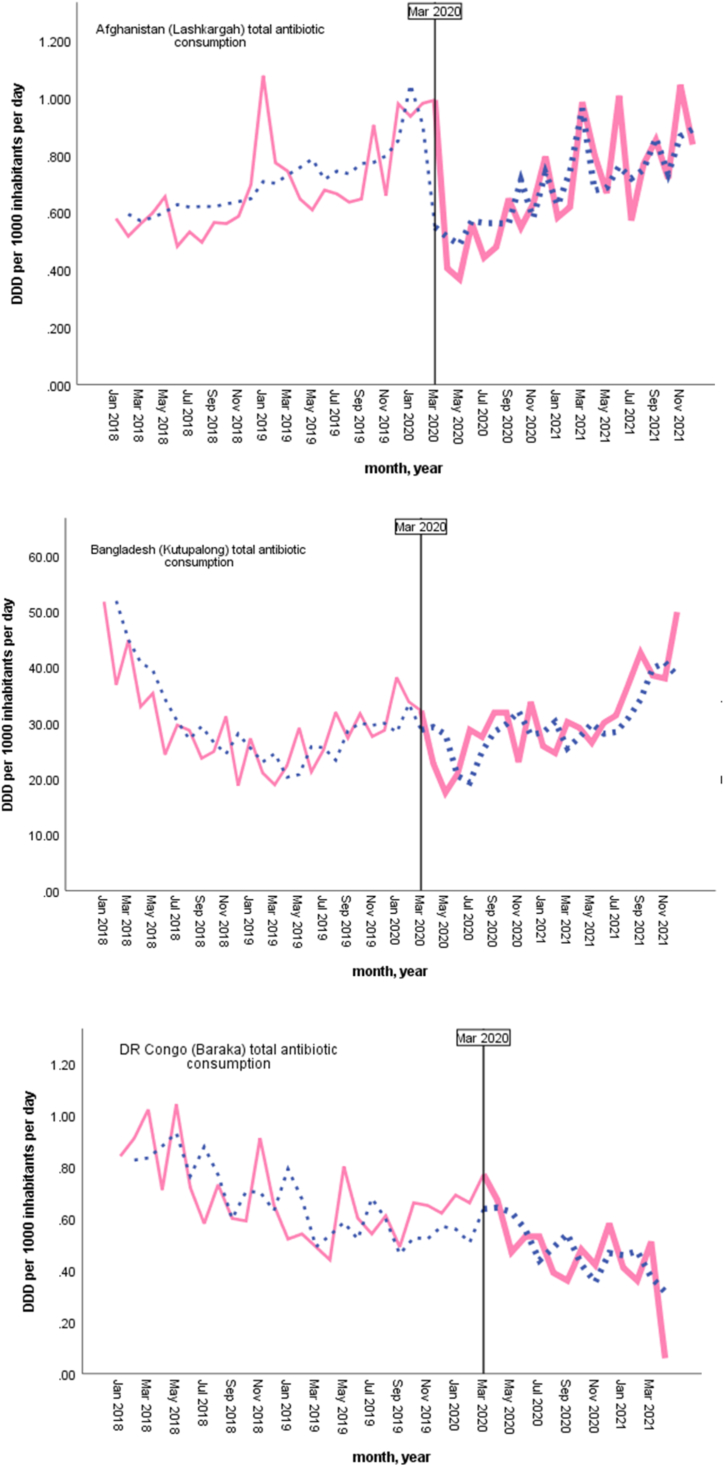

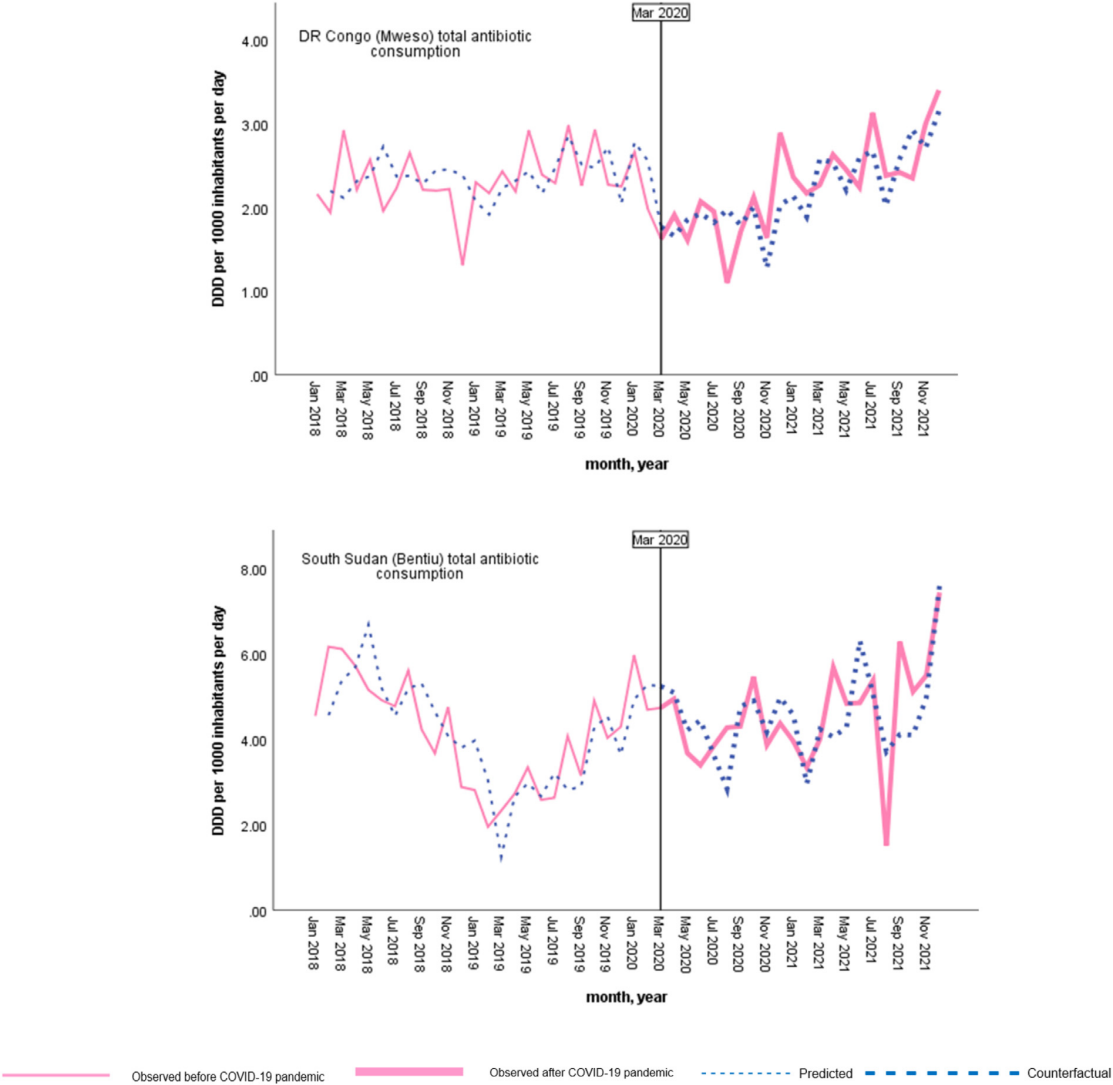
Trends in antibiotic consumption before and after COVID-19 pandemic based on ARIMA model results for each study setting.

Changes in total antibiotic consumption across the five humanitarian settings are presented in Table II, and ARIMA model graphs for each study setting are given in Figure 2. There were a variety of responses, and based on ITS-ARIMA models for each setting, no change occurred in total antibiotic consumption in South Sudan (Bentiu) and Bangladesh (Kutupalong) with the COVID-19 pandemic, including within the onset and first months of the pandemic. Similarly, no change occurred in total antibiotic consumption during the COVID-19 pandemic in DR Congo (Baraka). However, there was a small but statistically significant increase in antibiotic consumption at the onset of the pandemic (estimate = .274, p = 0.006), but not in the first months of the pandemic.

**Table II:** Changes in total antibiotic consumption (DDD per 1000 inhabitants per day) across five humanitarian settings

Humanitarian settings	March 2020		April–August 2020		March 2020–December 2021	
	Estimate	p	Estimate	p	Estimate	p
**Afghanistan (Lashkar Gah)**	.156	0.141	−.036	0.011	−.259	<0.001
**Bangladesh (Kutupalong)**	.448	0.323	−.807	0.342	−7.535	0.167
**DR Congo (Baraka)**^a^	.274	0.006	.009	0.649	.096	0.512
**DR Congo (Mweso)**	-.453	0.201	−.094	0.043	−.744	0.003
**South Sudan (Bentiu)**	.060	0.929	−.037	0.765	.086	0.925

a The setting had only antibiotic consumption data until April 2021.

An overall 0.74% decline (estimate = −.744, p = 0.003) was found in total antibiotic consumption for DR Congo (Mweso) setting during the COVID-19 pandemic, including a 0.09% decline (estimate = −.094, p = 0.043) in the first months of the pandemic. Within Afghanistan (Lashkar Gah), no change occurred in total antibiotic consumption with the onset of the pandemic, and following a 0.04% decline (estimate = −.036, p =0.011) was found in the fırst months. Overall, a 0.26% decline (estimate = −.259, p < 0.001) in total antibiotic consumption occurred for this setting during the COVID-19 pandemic.

Changes in consumption of Access group antibiotics and amoxicillin across the five humanitarian settings are presented in Table III. The availability of the Access antibiotics was to a large extent the same in all settings. Similar to total antibiotic consumption findings, no change occurred in Access group antibiotics including amoxicillin in South Sudan (Bentiu) and Bangladesh (Kutupalong). The initial increase in total antibiotic consumption observed in DR Congo (Baraka) was mainly driven by Access group antibiotics (estimate = .254, p <0.001). Despite this initial increase in Access group, however, no change occurred in amoxicillin within this setting during the COVID-19 pandemic.

**Table III:** Changes in Access group antibiotics and amoxicillin consumption (DDD per 1000 inhabitants per day) across five humanitarian settings

Humanitarian settings	March 2020		April–August 2020		March 2020–December 2021	
	Estimate	p	Estimate	p	Estimate	p
**Afghanistan (Lashkar Gah)**						
Access group antibiotics	.087	0.254	−.023	0.048	−.216	<0.001
Amoxicillin	.062	0.338	−.015	0.147	−.194	<0.001
**Bangladesh (Kutupalong)**						
Access group antibiotics	4.125	0.277	−.356	0.571	−4.461	0.253
Amoxicillin	4.616	0.115	−.566	0.280	−1.658	0.629
**DR Congo (Baraka)**^a^						
Access group antibiotics	.254	<0.001	−.005	0.764	.050	0.715
Amoxicillin	.099	0.289	.006	0.717	.063	0.592
**DR Congo (Mweso)**						
Access group antibiotics	−.513	0.087	−.076	0.112	−.701	0.005
Amoxicillin	−.483	0.103	−.126	0.004	−.588	0.036
**South Sudan (Bentiu)**						
Access group antibiotics	.191	0.609	−.069	0.417	.101	0.852
Amoxicillin	-.321	0.292	.032	0.729	−.402	0.481

a The setting had only antibiotic consumption data until April 2021.

Most of the decline in Access group antibiotics including amoxicillin was seen after the first few months of the pandemic. Overall, antibiotic consumption in DR Congo (Mweso) declined by 0.70% (estimate = −.701, p = 0.005) for Access group antibiotics, including a 0.59% (estimate = −.588, p =0.036) decline in amoxicillin antibiotic during the COVID-19 pandemic. There was no change in Access group antibiotics, including amoxicillin, at the onset of the pandemic, however, the consumption of amoxicillin declined by 0.13% (estimate = -.126, p =0.004) in the first months of the pandemic for this setting.

Overall, a 0.22% decline (estimate=−.216, p < 0.001) was found in the consumption of Access group antibiotics, including a 0.19% (estimate = −.194, p < 0.001) decline in amoxicillin consumption in Afghanistan (Lashkar Gah) during the pandemic. There was a small but statistically significant decline (estimate = −.023, p = 0.048) in Access group antibiotics in the first months of the pandemic for this setting.

Changes in consumption of Watch group antibiotics and azithromycin across all five humanitarian settings are presented in Table IV. The availability of the Watch antibiotics was to a large extent the same in all settings. Similarly, there was no change in consumption of Watch group antibiotics and azithromycin for South Sudan (Bentiu) and Bangladesh (Kutupalong) during COVID-19 pandemic, including at onset and during the first months of the pandemic.

**Table IV:** Changes in Watch group antibiotics and azithromycin consumption (DDD per 1000 inhabitants per day) across five humanitarian settings

Humanitarian settings	March 2020		April–August 2020		March 2020–December 2021	
	Estimate	p	Estimate	p	Estimate	p
**Afghanistan (Lashkar Gah)**						
Watch group antibiotics	.068	0.030	−.004	0.397	−.027	0.321
Azithromycin	.010	0.005	.000	0.821	−.001	0.752
**Bangladesh (Kutupalong)**						
Watch group antibiotics	−.088	0.971	−.587	0.140	−3.360	0.201
Azithromycin	−.260	0.867	−.309	0.359	.001	1.000
**DR Congo (Baraka)**^a^						
Watch group antibiotics	.035	0.349	.002	0.743	.013	0.745
Azithromycin	.009	0.533	.003	0.032	.013	0.051
**DR Congo (Mweso)**						
Watch group antibiotics	.141	0.220	−.019	0.207	−.055	0.584
Azithromycin	.064	0.310	−.016	0.083	−.148	<0.001
**South Sudan (Bentiu)**						
Watch group antibiotics	.137	0.737	−.071	0.169	−.281	0.403
Azithromycin	−.020	0.754	−.010	0.322	−.036	0.210

a The setting had only antibiotic consumption data until April 2021.

There was no overall change in the consumption of the Watch group antibiotics with COVID-19 pandemic for DR Congo (Baraka), despite a small increase (estimate = .003, p = 0.032) for azithromycin consumption in the first months of the pandemic. On the other hand, overall no change occurred in the consumption of the Watch group antibiotics in DR Congo (Mweso), and only the consumption of azithromycin declined by 0.15% (estimate = −.148, p<0.001) during the pandemic. Overall, no change was found in consumption of Watch group antibiotics including azithromycin with pandemic in Afghanistan (Lashkar Gah). However, there was only a 0.07% increase (estimate = .068, p =0.030) in the consumption of Watch group antibiotics, including a 0.01% increase (estimate=.010, p =0.005) in the consumption of azithromycin at the onset of COVID-19 pandemic.

## Discussion

This study aimed to assess the impact of the COVID-19 pandemic on antibiotic consumption trends across five geographically diverse humanitarian settings, all located in LMICs where the prevalence of multi drug resistant infections in patients admitted to hospitals remains a concern [54].

Overall, the results of this study suggest limited or no impact of COVID-19 pandemic on antibiotic consumption across five MSF's secondary healthcare hospitals. More specifically, no impact of the pandemic on antibiotic consumption was found for both South Sudan (Bentiu) and Bangladesh (Kutupalong) settings. Similarly, no overall impact was found in DR Congo (Baraka), despite an initial increase in antibiotic consumption at the onset of the pandemic, which was mainly driven by an increase in Access group antibiotics. However, there was a small increase in consumption of azithromycin (Watch group antibiotic) during the first months of the pandemic for this setting. Conversely, overall antibiotic consumption declined during the COVID-19 pandemic in DR Congo (Mweso), and the decline occurred in Access group antibiotics, including amoxicillin, and the Watch group antibiotic, azithromycin. No change was found to have occurred, including in the consumption of Access and Watch groups antibiotics, at the onset of COVID-19 pandemic in this setting. Like DR Congo (Mweso), Afghanistan (Lashkar Gah) also experienced a decline in Access group antibiotics, including amoxicillin, while an increase was found in Watch group antibiotics, including azithromycin, at the onset of COVID-19 pandemic.

Khouja's study [39] reported an initial increase in antimicrobial consumption (antibiotics contributed most) during COVID-19 pandemic using data of 66 countries. However, considering our study's findings an initial increase was only found in total antibiotic consumption of DR Congo (Baraka). Regarding studies from LMICs on antibiotic consumption during the pandemic, more specifically, the findings from Afghanistan (Lashkar Gah), for example, are consistent with those in Jordan reported by Al-Azzam's study [21], which indicated a decrease in total antibiotic consumption despite the increase of Watch group antibiotics. That said, Afghanistan (Lashkar Gah)'s results differed from its neighbouring country, Pakistan, which reported higher antibiotic consumption in COVID-19 patients admitted to the five hospitals in Punjab province, compared to pre-pandemic period [55]. While both South Sudan (Bentiu) and Bangladesh (Kutupalong) seemed to have withstood the pandemic's impact on antibiotic consumption, Molla [56] and Parveen's [57] studies from Bangladesh reported persistent challenges with regards to overuse of antibiotics, including high antibiotic prescription and self-medication among COVID-19 patients.

The findings of this study suggest that the humanitarian settings included in this study managed to fare the pandemic's impact on antibiotic consumption observed elsewhere. However, there might also be hidden factors that lead to these findings. Confirmed COVID-19 case numbers were not available for the study settings during the study period, as only Bangladesh (Kutupalong) and South Sudan (Bentiu) had data on admission/consultations of suspected COVID-19 cases, and that limits the interpretation of our study findings. Moreover, at the start of the pandemic, there was limited capacity for laboratory testing for COVID-19, and that might hinder actual COVID-19 case numbers [58] for these low-resourced settings. On the other hand, data on admission/diagnosis of acute respiratory infections (ARI) were available during the same period for the five settings. Similar to the decline in total antibiotic consumption in Afghanistan (Lashkar Gah), ARI admissions/diagnosis had the lowest proportion in 2020 for this setting. In addition, similarly the proportion of ARI admissions/diagnosis declined in 2020 compared to previous year in DR Congo (Baraka) hospital. Restrictions on mobility, particularly in the beginning of the pandemic [59] might have an impact on patient admissions to the hospitals in Afghanistan (Lashkar Gah) and DR Congo (Baraka).

Moreover, despite overall total antibiotic consumption declining in DR Congo (Mweso), the proportion of ARI admission increased, and the proportion of ARI diagnosis remained stable in 2020 compared to the previous year. Related to the settings that pandemic had no impact on antibiotic consumption, the proportion of both ARI admission/diagnosis increased for Bangladesh (Kutupalong) in 2020 compared to the previous year. Differently, despite the increase in the proportion of ARI admissions, the proportion of ARI diagnosis decreased for South Sudan (Bentiu) in 2020, compared to the previous year. In addition, many supply disruptions and shortages during COVID-19 pandemic have been reported [60]. Shortages in antibiotic supply might explain some findings including decreased total antibiotic consumption but stable proportions of ARI diagnosis.

Taken together, all these factors/efforts might explain why the findings of this study differ from global trends in antibiotic consumption during the initial stages of the COVID-19 pandemic [28]. However, further research with health professionals responsible for antibiotic stewardship activities in each context would improve and strengthen our understanding of why different antibiotic consumption trends were observed across the five contexts.

The current study is not without limitations. First, as a retrospective analysis of observational data, the true impact of the COVID-19 pandemic on antibiotic consumption is difficult to ascertain. However, in order to mitigate this limitation, ITS/ARIMA model was used as it is considered a more robust design to establish causality within observational data where randomised controlled trials (RCTs) are not feasible [52]. Lack of information about COVID-19 diagnoses in most of the study hospitals makes it difficult to establish true burden of COVID-19 in our study settings and therefore to conclude how it might have affected antibiotic consumption. In addition, antibiotic consumption data gathered from consumption tools do not represent the real inpatient consumption, however represent the volumes of antibiotics that have been dispensed to the hospital departments. Finally, while the data indicates changes in antibiotic consumption over time, we cannot evaluate the appropriateness of antibiotic use for the same time period.

## Conclusion

Overall, the results of this study suggest no impact of COVID-19 pandemic on total antibiotic consumption in Bangladesh (Kutupalong), South Sudan (Bentiu) and DR Congo (Baraka). However, overall a decline has been found in total antibiotic consumption during COVID-19 pandemic in Afghanistan (Lashkar Gah) and DR Congo (Mweso). Monitoring antibiotic consumption is considered key to tackling AMR. However, further studies are needed to understand the mechanisms that underpin this study's findings, and to understand whether these antibiotic consumption trends are replicated in other humanitarian settings, more broadly, so as to better guide future practices and policies in order to optimise antibiotic use in the humanitarian settings.

## Author contributors

TY, KC, KS, JG and FV conceived the study. TY conducted analyses and drafted the initial manuscript. KC, KS, JG and FV revised the manuscript. All authors contributed to the final manuscript. TY is responsible for the overall content and publication.

## Funding

None.

## Conflict of interest statement

None.

## Patient consent for publication

Not required.

## Ethics approval

Ethical approval for this study was obtained from the Health Policy & Management/Centre for Global Health Research Ethics Committee, Trinity College Dublin (application number: 03/2022/02) and MSF Research Review Committee.
